# Supplementary material for: A bacteriocin expression platform for targeting pathogenic bacterial species
Source: Nat Commun. 2024 Jul 27;15:6332. doi: 10.1038/s41467-024-50591-8 (PMC11283563; doi:10.1038/s41467-024-50591-8)
Supplement: Supplementary file 1 — Supplementary information [file 41467_2024_50591_MOESM1_ESM.pdf]

Supplementary Information:
A bacteriocin expression platform for targeting pathogenic bacterial species

Jack W. Rutter*, Linda Dekker*, Chania Clare, Zoe F. Slendebroek, Kimberley A. Owen, Julie A.K. McDonald, Sean P. Nair, Alexander J.H. Fedorec, and Chris P. Barnes

Contents

1 Extended materials & methods **3**

1.1 Strains & Plasmids . . . 3

1.2 Synthetic bacteriocin assays . . . 3

1.3 Live/Dead cell assays . . . 3

1.4 Peptide concentration by ammonium sulphate precipitation . . . 6

1.5 Tricine SDS-PAGE . . . 6

1.6 Gel activity assay . . . 7

1.7 Inducible assays . . . 7

1.8 GspD knockout assays . . . 7

1.9 Anaerobic inhibition zone assays . . . 7

1.10 Multiplexed library construction . . . 8

1.11 Colony-counting assays . . . 8

1.12 Gompertz growth curve fitting . . . 8

1.13 Software versions . . . 8

2 Pairwise mathematical models and Bayesian analysis **9**

2.1 Baseline model: linear Lotka-Volterra . . . 9

2.2 Saturated Lotka-Volterra with reusable bacteriocin . . . 9

2.3 Joint modelling of experiments . . . 9

2.4 Models explored . . . 10

2.5 Statistical model . . . 10

2.6 Fitting procedure . . . 11

2.7 Estimated posteriors for Model 5 . . . 11

3 Extended results **12**

References **30**

1 Extended materials & methods

1.1 Strains & Plasmids

A full list of all reagents used in the manuscript are provided in Supplementary Table 1. All strains and plasmids used in this study are given in Supplementary Table 2. The DNA sequences used for the secretion signal and bacteriocin parts are given in Supplementary Table 3.

1.2 Synthetic bacteriocin assays

Assay plates were prepared with 30 ml of sterile BHI 1.5% agar in 90 mm petri dishes. Plates were allowed to set before 2 μl of overnight culture for the target strain was diluted into 150 μl of fresh BHI and spread on the surface of the assay plate. Once dry, loading wells were cut into the agar using a sterile glass Pasteur pipette. 50 μl of desired synthetic bacteriocin concentrations were then added to the loading wells and the plates incubated for 18 hours at 37°C. Images were then collected and processed as described in the main text.

Synthetic bacteriocin timecourses were performed on *E. faecalis* mono-cultures. Overnight *E. faecalis* cultures were adjusted to $OD_{700} = 1$ and then diluted 1:100 in fresh BHI media. The diluted cultures were then supplemented with the desired bacteriocin concentrations and 120 μl added to each well of a 96 well plate. The growth curves were then recorded by measuring OD_{700} absorbance every 20 minutes for a total of 48 hours.

1.3 Live/Dead cell assays

The live/dead cell assay was performed using the LIVE/DEAD BacLight Bacterial Viability kit (Thermo Scientific, USA). In brief, *E. faecalis* were inoculated from glycerol stocks and grown in BHI media for approximately 18 hours at 37°C with shaking. Cultures were then diluted 1:20 and left to grow to an OD_{700} of 0.4. Cells were then split into 1 ml aliquots for each condition. For the 'none', 'EntA' and 'EntB' treatment groups cultures were supplemented with either 0 or 4 μg/ml of the respective bacteriocin and incubated for a further hour. The ethanol treated 'negative' control group was created as per the manufacturers instructions. After the one hour incubation all cultures were centrifuged and washed twice with PBS (Lonza, Belgium). The washed cells were then resuspended in PBS at 10x the original culture volume. Diluted cultures were supplemented with the SYTO-9 and propidium iodide dyes at a final ratio of 2:1 up to a total concentration of 3 μl/ml. Microscope slide agarose pads were prepared by adding 500 μl of 1.5% agarose to clean slides, following the instructions given by Skinner *et al* (2013)[3]. Five μl of culture from the selected treatment group was added to each agarose pad. After one minute, the agarose pads were covered by clean cover slips and sealed with clear acrylic polish. The prepared samples were then imaged at 40x magnification with an Olympus Widefield 1X81 microscope (Olympus, Japan). Images were taken in the following channels; brightfield, TRITC (red) for propidium iodide stain (ex = 535nm, em = 617nm) and FITC (green) for SYTO-9 stain (ex = 485nm, em = 498nm), with a 50 ms exposure time. Images were collected and saved with the opensource 'Micro-Manager' software in Fiji[4].

All image analysis was performed in Fiji software. Collected images were first split into individual channels and converted to 8-bit. The FITC and TRITC images were then manually thresholded to identify the stained areas in the red and green channels. The thresholded areas were then measured

Supplementary Table 1: A full list of the chemical and reagents used within this study.

Item	Supplier	Product number
16.5% Mini-PROTEAN Tris/Tricine Precast Gel	Bio-Rad	Bio-Rad
Agar	Millipore	5039
Ammonium Sulfate	Sigma Aldrich	09978
Ampicillin Sodium Salt	Sigma Aldrich	A9518
Anhydrotetracycline Hydrochloride	Thermo Fisher Scientific	J66688-MA
BbsI-HF	New England Biolabs	R3539S
Brain Heart Infusion Broth	Oxoid	CM1135
Breathe-easy Sealing Membrane	Electron Microscopy Sciences	70536-10
BsaI-HF v2	New England Biolabs	R3733L
Gentamycin Sulfate Salt	Sigma Aldrich	G3632
Kanamycin Sulfate	Fluorochem	M02038
L-(+)-Arabinose	Alfa Aesar	A11921
LB Broth	Sigma Aldrich	L3022
LIVE/DEAD BacLight Bacterial Viability Kit	Thermo Fisher Scientific	L7012
Microplate 96-Well PS μClear Black Med. Binding	Greiner Bio-One	655096
Microscope Slides T/F Ground 0.8-1.0mm	Fisher Scientific	1156-2203
Monarch Plasmid Miniprep Kit	New England Biolabs	T1010L
NEB®5-α Competent *E. coli* (High Efficiency)	New England Biolabs	C2987U
NEB®Express Competent *E. coli* (High Efficiency)	New England Biolabs	C2523H
NuPAGE Sample Reducing Agent (10X)	Thermo Fisher Scientific	NP0004
Precision Plus Protein Dual Xtra Prestained Protein Standard	Bio-Rad	1610377
Streptomycin Sulfate	Fisher Scientific	Fisher Scientific
T4 DNA Ligase (HC)	Promega	M179A
T4 DNA Ligase Buffer (10X)	Promega	C126B
Tris-Acetate SDS Running Buffer (20X)	Novex by Life Technologies	LA0041
Tris-Glycine SDS Sample Buffer (2X)	Novex by Life Technologies	LC2676

Supplementary Table 2: Strains and plasmids used within this study (RBS = ribosome binding site, CDS = coding DNA sequence).

**Strain**	**Description**	**Source**
E. coli NEB®5-α	Commercial cell line used for cloning	New England Biolabs
E. coli NEB®Express	Commercial cell line used for protein expression	New England Biolabs
E. coli Nissle 1917	Commensal strain of *E. coli*	Prof. Ian Henderson (Uni. of Birmingham, UK)
E. coli BW25113	Parent strain of the Keio knockout collection	Keio Collection[1]
E. coli JW5707	BW25113 $\Delta gspD$ knockout strain	Keio Collection
E. faecalis DSM25700	*E. faecalis* strain	DSMZ collection
E. faecium NCTC12202	Vancomycin-resistant isolate of *E. faecium*	Dr Julie MacDonald (Imperial College, UK)
E. coli sAJM.1506	Engineered $tetR^{+}$ marionette strain	[2]
**Plasmid**	**Description**	**Source**
pMalE-EntA	J23106 promoter, BCD12 RBS, MalE-EntA CDS, B0015 terminator, DVK_AF vector, Kan^R	This study
pMalE-EntB	J23106 promoter, BCD12 RBS, MalE-EntB CDS, B0015 terminator, DVK_FG vector, Kan^R	This study
pMalE-EntAB	pMalE-EntA, pMalE-EntB, DVA_AG vector, Amp^R	This study
pOmpA-EntA	J23106 promoter, BCD12 RBS, OmpA-EntA CDS, B0015 terminator, DVK_AF vector, Kan^R	This study
pOmpA-EntB	J23106 promoter, BCD12 RBS, OmpA-EntB CDS, B0015 terminator, DVK_FG vector, Kan^R	This study
pOmpA-EntAB	pOmpA-EntA, pOmpA-EntB, DVA_AG vector, Amp^R	This study
pPhoA-EntA	J23106 promoter, BCD12 RBS, PhoA-EntA CDS, B0015 terminator, DVK_AF vector, Kan^R	This study
pPhoA-EntB	J23106 promoter, BCD12 RBS, PhoA-EntB CDS, B0015 terminator, DVK_FG vector, Kan^R	This study
pPhoA-EntAB	pPhoA-EntA, pPhoA-EntB, DVA_AG vector, Amp^R	This study
pPM3-EntA	J23106 promoter, BCD12 RBS, PM3-EntA CDS, B0015 terminator, DVK_AF vector, Kan^R	This study
pPM3-EntB	J23106 promoter, BCD12 RBS, PM3-EntB CDS, B0015 terminator, DVK_FG vector, Kan^R	This study
pPM3-EntAB	pPM3-EntA, pPM3-EntB, DVA_AG vector, Amp^R	This study
pFlopR-mCherry	p15A origin, J23101 promoter, Elowitz strong RBS, mCherry2, ECK120033736 terminator, $Strep^R$	This study
pFlopR-GFP	J23106 promoter, B0032m RBS, SfGFP CDS, B0015 terminator, DVK_AF vector, Kan^R	This study
pAraBAD-GFP	ParaBAD/araC promoter, B0032m RBS, sfGFP CDS, B0015 terminator, DVK_AF, Kan^R	This study
pAraBAD-PM3EntA	ParaBAD/araC promoter, B0032m RBS, PM3-EntA CDS, B0015 terminator, DVK_AF, Kan^R	This study
pTet-GFP	R0040_AB, B0032m RBS, sfGFP CDS, B0015 terminator, DVK_AF, Kan^R	This study
pTet-PM3EntA	R0040_AB, B0032m RBS, PM3-EntA CDS, B0015 terminator, DVK_AF, Kan^R	This study
pPM3-EntA(amp)	J23106 promoter, BCD12 RBS, PM3-EntA CDS, B0015 terminator, DVA_AE vector, Amp^R	This study
pPM3-EntA-GspD	J23106 promoter, BCD12 RBS, PM3-EntA CDS, B0015 terminator, J23106 promoter, B0032m RBS, GspD CDS, B0015 terminator, DVA_AF vector, Amp^R	This study

Supplementary Table 3: DNA sequences used to create the secretion signal and bacteriocin parts (**bold** indicates the stop codon).

**Part**	**DNA sequence** (5' - 3')
PM3	AAATACCTGCTGCCGACCGCTGCTGCTGGTCTGCTGCTCCTCGCTG CCCAGCCGACGATGGCC
OmpA	AAAAAGACAGCTATCGCGATTGCAGTGGCACTGGCTGGTTTCGCTA CCGTAGCGCAGGCC
PhoA	AAACAAAGCACTATTGCACTGGCACTCTTACCGTTACTGTTTACCCC TGTGACAAAAGCC
MalE	AAAATAAAAACAGGTGCACGCATCCTCGCATTATCCGCATTAACGAC GATGATGTTTTCCGCCTCGGCTCTCGCC
Ent A	ACCACTCATAGCGGTAAGTATTACGGAAATGGAGTTTACTGTACCAA AAATAAATGCACCGTTGATTGGGCTAAAGCGACAACTTGTATCGCT GGTATGTCTATCGGCGGGTTCTTAGGGGGTGCCATTCCAGGCAAAT GC**TAA**
Ent B	GAAAACGACCACAGAATGCCCAACGAGTTGAATCGCCCTAACAATC TTAGCAAAGGGGGAGCCAAATGCGGCGCGGCGATTGCAGGTGGA CTTTTCGGGATACCGAAAGGACCGCTGGCTTGGGCCGCTGGATTAG CGAATGTTTACTCAAAATGTAAC**TAA**

and the ratio of red:green stained areas used to estimate the percentage of dead cells in each treatment group, with a maximum threshold of 100%. Statistical analysis between treatment groups was performed in R, using an unpaired t-test. A p-value of less than 0.05 was deemed to show a significant difference between treatment groups.

1.4 Peptide concentration by ammonium sulphate precipitation

Bacteriocin expressing strains were inoculated into 40 mL LB medium + antibiotics from glycerol stocks and incubated for ~ 16 hr at 37°C with shaking. *E. coli* cultures were pelleted by centrifugation at 3,400g for 10 min and supernatants poured into 100 ml flasks containing stir bars. Ammonium sulphate salt was then slowly added to each 40 ml sample to reach 70% saturation concentration at 4°C. Samples were mixed by rotation at 4°C overnight. The following day, proteins were pelleted by centrifugation at 11,000g at 4°C for 15 min. Supernatant was removed, and the pelleted precipitate was resuspended in 300 μl sterile deionised water and stored at -20°C.

1.5 Tricine SDS-PAGE

A total volume of 12 μl of the concentrated supernatants were combined with 3 μl NuPAGE Reducing Agent (10x), and 15 μl Novex^{TM} Tris-Glycine SDS Sample Buffer (2x). 30 μl samples were loaded onto a precast 16.5% Tris-Tricine gel (Bio-Rad). Then, 10 μl of Precision Plus Protein^{TM} Dual Xtra Prestained Protein Standard (Bio-Rad) was loaded into the first well. The gel was run for 10 minutes at 65 mA, followed by 30 mA until complete in Novex^{TM} Tricine SDS Running Buffer. It was then rinsed in deionised water.

1.6 Gel activity assay

Molten 1% BHI agar was inoculated with 0.5 μl overnight culture of *E. faecium* per ml of agar, gently mixed by inversion, then poured into a one well plate and allowed to solidify. The rinsed Tricine SDS-PAGE gel was placed on the agar, incubated overnight at 37°C and was imaged the following day.

1.7 Inducible assays

The pAraBAD-GFP and pAraBAD-PM3EntA circuits were transformed and characterised in *E. coli* NEB®Express. The pTet-GFP and pTet-PM3EntA circuits were transformed and characterised in the marionette *E. coli* strain (sAJM.1506), which has genomic expression of the TetR transcription factor.

GFP sensor versions of both circuits were characterised in liquid culture. The strains were inoculated from glycerol stocks and grown in BHI media for 18 hours at 37°C with shaking. The cultures were then adjusted to a starting OD_{700} of 0.01. Adjusted strains were supplemented with the desired L-arabinose or aTc concentration and added to the wells of a 96-well plate (up to a total volume of 120 μl). Plates were then incubated for 16 hours at 37°C with shaking (300 rpm, 2 mm orbital), in a Tecan Spark plate reader (Tecan, USA). Measurements for OD_{700} and GFP fluorescence (excitation: 488/20 nm, emission: 530/20 nm, gain: 70) were collected at the final timepoint. A Hill function was used to fit the measured GFP values. The fitting was performed using the 'nls' function in R, using equation (1):

$$f = f_{min} + (f_{max} - f_{min}) \cdot \frac{[x]^n}{K_d^n + [x]^n} \tag{1}$$

Where f is the observed GFP, f_{min} is the minimum fitted GFP value, f_{max} is the maximum fitted GFP value, $[x]$ is the inducer concentration, K_d is the threshold sensitivity and n is the cooperativity.

Solid-culture inhibition assays of the pAraBAD-PM3EntA and pTet-PM3EntA strains were performed as detailed in the main text, except for the addition of inducers in the BHI agar plates to the desired concentrations.

1.8 GspD knockout assays

The Δ*gspD* knockout strain was purchased from the Keio knockout collection[1] and transformed with the desired bacteriocin expression plasmid. Inhibition zone assays were performed as detailed in the standard solid culture characterisation protocol given in the main text.

1.9 Anaerobic inhibition zone assays

Anaerobic inhibition zones assays were performed as described in the main text, with two modifications. Both the 18 hour incubation of inoculated strains and the final 18 hour incubation of the assay plate were performed in an anaerobic environment (10% CO_2, 10% H_2, 80% N_2). Anaerobic conditions were maintained with a miniMACS anaerobic workstation (Don Whitley Scientific, UK).

1.10 Multiplexed library construction

Multiplex reactions using the CIDAR MoClo assembly method were performed in 20 μl volumes with 60 fmol of each part type [5]. The MoClo reaction mix was transformed into *E. coli* NEB®5α competent cells (New England Biolabs, USA) according to the manufacturer's instructions. Individual colonies (x100) were stabbed into BHI plates that contained an *E. faecalis* lawn (see main text, solid culture characterisation). Plasmid DNA was extracted from ten isolates with the largest inhibition zones (NEB Monarch® Plasmid Miniprep Kit) and sent for Sanger sequencing so the parts could be identified. Six unique PM3-EntA producing constructs were identified, these are given in Figure S15C.

1.11 Colony-counting assays

Co-cultures of the desired strains were set up following the standard liquid co-culture characterisation protocol given in the main text. After 8 hours the timecourse was paused and a 1 μl sample of each culture was serially diluted in fresh BHI media. 5 μl of each dilution was plated on an *E. faecalis* selection plate (30 ml of BHI media supplemented with 10 μg/ml gentamycin). These plates were then incubated for approximately 18 hours at 37°C and the resultant growth used to estimate colony forming units.

1.12 Gompertz growth curve fitting

A Gompertz growth model was used to fit the mono-culture growth curves shown in Figure S7. The fitting was performed using the 'nls' function in R, using equation (2):

$$y(t) = y_0 + (A - y_0) \cdot exp(-exp(\mu(\lambda - t)/(A - y_0) + 1)) \tag{2}$$

Where t is time, y the measured optical density, y_0 the optical density at $t = 0$, μ the predicted growth rate, λ the predicted lag time and A the predicted carrying capacity. These parameters are depicted in Figure S7B.

1.13 Software versions

Supplementary Table 4 provides a list of all software versions used within this manuscript.

Supplementary Table 4: A full list of software versions used within this study.

Software	Version
Adobe Illustrator	28.5
Fiji (ImageJ2)	2.9.0/1.53t
Motif Recording Software	5.2.0 beta27-0jrs1
R	4.1.2
RStudio	2022.07.2
Spark Control	2.3

2 Pairwise mathematical models and Bayesian analysis

In the following x_1 refers to *E. coli* and x_2 refers to *E. faecalis.* Since we don't observe the dynamics of the bacteriocins directly we revert to pairwise models to capture the behaviour.

2.1 Baseline model: linear Lotka-Volterra

Control (no bacteriocin production)
Here we set M_{21} to zero and have non zero M_{12}, motivated by the observation that the levels of *E. faecalis* seem independent of whether the control *E. coli* is present.

$$\dot{x_1} = x_1\mu_{Ec} - x_1M_{11}x_1 - x_1M_{12}x_2$$
$$\dot{x_2} = x_2\mu_{Ef} - x_2M_{22}x_2$$

Bacteriocin production
We assume the same functional form for EntA and EntB producing *E. coli.* Now we include a non zero (negative) M_{21} to capture bacteriocin action on *E. faecalis.*

$$\dot{x_1} = x_1\mu^s_{Ec} - x_1M^s_{11}x_1 - x_1M_{12}x_2$$
$$\dot{x_2} = x_2\mu_{Ef} - x_2M_{22}x_2 - x_2M^s_{21}x_1$$

where $s = \{A, B, AB\}$ denotes the *E. coli* strains expressing bacteriocins EntA, EntB and EntAB respectively.

2.2 Saturated Lotka-Volterra with reusable bacteriocin

Here we modify the negative interaction due to bacteriocin action to take a saturated form. Although this is a common pairwise model it can also be derived from the full mechanistic model assuming that either x_1 grows faster than x_2 or the exchange molecule produce by x_1 (the bacteriocin) is reusable [6]. Since *E. faecalis* (x_2) grows faster than *E. coli* (x_1) we can assume the model captures the second scenario.

Bacteriocin production

$$\dot{x_1} = x_1\mu^s_{Ec} - x_1M^s_{11}x_1 - x_1M_{12}x_2$$
$$\dot{x_2} = x_2\mu_{Ef} - x_2M_{22}x_2 - \frac{x_2M^s_{21}x_1}{K^s + x_1}$$

where $s = \{A, B, AB\}$ denotes the *E. coli* strains expressing bacteriocins EntA, EntB and EntAB respectively.

2.3 Joint modelling of experiments

We simultaneously fit the mono- and co-culture data, comprising nine separate timecourse experiments (see Supplementary Table 5).

We assume that:

Supplementary Table 5: Data used for model fitting. Each coculture data set contains two timeseries, j=1 (*E. coli*), j=2 (*E. faecalis*).

Symbol	Type	Description
y_{1j}	co-culture	*E. coli* control vs *E. faecalis*
y_{2j}	co-culture	*E. coli* PM3-EntA vs *E. faecalis*
y_{3j}	co-culture	*E. coli* PM3-EntB vs *E. faecalis*
y_{4j}	co-culture	*E. coli* PM3-EntAB vs *E. faecalis*
y_5	mono-culture	*E. coli* control
y_6	mono-culture	*E. coli* PM3-EntA
y_7	mono-culture	*E. coli* PM3-EntB
y_8	mono-culture	*E. coli* PM3-EntAB
y_9	mono-culture	*E. faecalis*

- The mutational burden of bacteriocin production is the same for EntA, EntB and EntAB but different to the wildtype. This means we consider three growth rates: μ_{Ec} for the *E. coli* wildtype, μ'_{Ec} for the *E. coli* bacteriocin secretion strains and μ_{Ef} for *E. faecalis.*
- M_{22} is the same in each co-culture experiment. That is, *E. faecalis* self interaction doesn't depend on bacteriocin production.
- The interaction between *E. faecalis* on *E. coli*, M_{12}, to be the same across experiments.

We explored how self interaction of *E. coli*, M_{11}, changes between control and engineered strains using Bayesian model selection.

2.4 Models explored

Model 1 : Linear Lotka-Volterra with two self interaction terms: M_{11} for all *E. coli* strains and and M_{22} for *E. faecalis.*

Model 2 : Linear Lotka-Volterra with five self interaction terms: M_{22} for *E. faecalis* and four different M_{11} for the individual *E. coli* strains, M_{11}, M_{11}^A, M_{11}^B, M_{11}^{AB}.

Model 3 : Linear Lotka-Volterra with three self interaction terms: M_{22} for *E. faecalis*, M_{11} for the *E. coli* wildtype and M'_{11} for the engineered *E. coli* strains ($M_{11}^A = M_{11}^B = M_{11}^{AB} = M'_{11}$).

Model 4 : Saturated Lotka-Volterra (reusable bacteriocin) with two self interaction terms: M_{11} for all *E. coli* strains and and M_{22} for *E. faecalis.*

Model 5 : Saturated Lotka-Volterra (reusable bacteriocin) with three self interaction terms: M_{22} for *E. faecalis*, M_{11} for the *E. coli* wildtype and M'_{11} for the engineered *E. coli* strains ($M_{11}^A = M_{11}^B = M_{11}^{AB} = M'_{11}$).

2.5 Statistical model

Likelihood We assume a LogNormal likelihood, which is equivalent to fitting on the log scale with normally distributed errors:

$$y(t) \sim \text{LogNormal}(\log(x(t)), \sigma)$$

where $y(t)$ is the observed data of each species and $x(t)$ is corresponding output from the ODE model. This approach is used for variables that are strictly positive and when transformed back to the natural scale leads to multiplicative errors. This kind of likelihood has also been used extensively to model microbial dynamics (for examples [7, 8]).

Priors We chose wide and relatively uninformative priors for the inference. We constrained all interaction terms to be negative as expected for competitive interactions.

$$\begin{aligned}
\mu &\sim \mathrm{U}(0, 0.05) \\
M_{ii} &\sim \mathrm{U}(-0.2, 0) \\
M_{12} &\sim \mathrm{U}(-0.1, 0) \\
M_{21} &\sim \mathrm{U}(-0.2, 0) \\
x(t=0) &\sim \mathrm{LogNormal}(\log(0.01), 1.0) \\
\sigma &\sim \mathrm{LogNormal}(-1, 1) \\
K &\sim N(0.2, 0.1)\mathbb{I}(x > 0)
\end{aligned}$$

We chose a truncated Normal distribution for K as we expect the bacteriocin to be active at lower rather than higher concentrations. The posterior distributions were found to be robust to changes in the prior distributions.

2.6 Fitting procedure

We used the Rstan interface [9] to the Stan probabilistic programming language [10] to fit the model. All models were fit using 4 chains and convergence verified using the criterion $\hat{R} < 1.1$. Each chain contained 1000 samples, with the first 500 discarded. The final 500 samples of all four chains were combined for the final posterior estimate. All the code for the fitting is included in the Zenodo repository.

2.7 Estimated posteriors for Model 5

Supplementary Table 6 gives the list of parameters and the estimated posterior values for Model 5. Note that for clarity we have omitted the inferred values of the initial conditions. These can be accessed using the associated R code.

Supplementary Table 6: Estimated parameters in Model 5 and their 0.95 credible regions.

Symbol	Description	Units	$q_{0.025}$	$q_{0.5}$	$q_{0.975}$
μ_{Ec}	Growth rate of wildtype *E. coli*	min^{-1}	0.0309	0.0336	0.0364
μ'_{Ec}	Growth rate of bacteriocin secreting *E. coli*	min^{-1}	0.0219	0.023	0.0243
μ_{Ef}	Growth rate of *E. faecalis*	min^{-1}	0.0338	0.0365	0.0392
M_{11}	*E. coli* (wildtype) self interaction	min^{-1} OD^{-1}	-0.0833	-0.0655	-0.052
M'_{11}	*E. coli* (secretion) self interaction	min^{-1} OD^{-1}	-0.0454	-0.0389	-0.0331
M_{22}	*E. faecalis* self interaction	min^{-1} OD^{-1}	-0.1046	-0.0889	-0.0754
M_{12}	Interaction of *E. faecalis* on *E. coli*	min^{-1} OD^{-1}	-0.0597	-0.0492	-0.0395
M_{21}^{A}	Interaction of Ec-EA on *E. faecalis*	min^{-1}	-0.0258	-0.0225	-0.0193
M_{21}^{B}	Interaction of Ec-EB on *E. faecalis*	min^{-1}	-0.0415	-0.0241	-0.0103
M_{21}^{AB}	Interaction of Ec-EAB on *E. faecalis*	min^{-1}	-0.0308	-0.0242	-0.02
K^{A}	Half-maximum constant associated with EntA	OD	1e-04	0.0012	0.0051
K^{B}	Half-maximum constant associated with EntB	OD	0.143	0.292	0.4618
K^{AB}	Half-maximum constant associated with EntAB	OD	0.0037	0.0155	0.074
σ	Measurement noise on the log scale	dimensionless	0.2875	0.3132	0.3434

3 Extended results

Figure S1 shows the screening of synthetic EntA and EntB activity against *E. faecalis* and *E. coli* NEB®express cells.

Figure S2 summarises the results from the live/dead assay performed to assess the bactericidal activity of synthetic EntA and EntB bacteriocins.

Figure S3 gives predictions of the secretion signal cleavage sites.

Figure S4 shows Tricine SDS-PAGE activity gel results, confirming the production of EntA and EntB from the eLBP strains.

Figure S5 summarises the FlopR process used for predicting individual strain growth in co-culture.

Figure S6 provides co-culture heatmaps and timecourses, exploring the affects of initial culture density and seeding ratio on final co-culture composition.

Figures S7 show the mono-culture growth curves and Gompertz parameter fits for all strains used in this study.

Figure S8 shows 48 hour timecourses of *E. faecalis* grown in co-culture with the control, PM3-EntA, PM3-EntB and PM3-EntAB secreting strains.

Figure S9 gives a comparison of all the models tested within this study and provides the posterior distributions of all the parameters fitted within the Lokta-Volterra model.

Figure S10 shows a comparison of experimental versus simulated ratios for the *E. coli* PM3-EntA and *E. faecalis* co-cultures.

Figure S11 the design and characterisation of arabinose-inducible and aTc-inducible bacteriocin producing strains, based on the commonly used ParaBAD and PTet inducible promoters.

Figure S12 shows the antimicrobial activity of the PM3-EntA bacteriocin constructs (with and without plasmid expression of the *gspD* gene), when expressed from the Δ*gspD* knockout (JW5707) and $gspD^{+}$

(BW25113) host strains.

Figure S13 summarises the GFP fluorescence measurements that were used to calculate the bystander strain OD in the 3-strain co-culture assays.

Figure S14 gives the results of inhibition zone assays for the PM3-bacteriocin expressing strains against *E. faecalis*, when grown in anaerobic conditions.

Figure S15 shows the results of a multiplex assembly reaction, used to create a range of PM3-EntA expressing eLBP strains. Using a mixture of promoters and RBS parts, strains with differing antimicrobial activity and growth rates were created.

Figure S16 provides estimates of the *E. faecalis* colony counts taken directly from the FlopR co-cultures after 8 hours of growth.

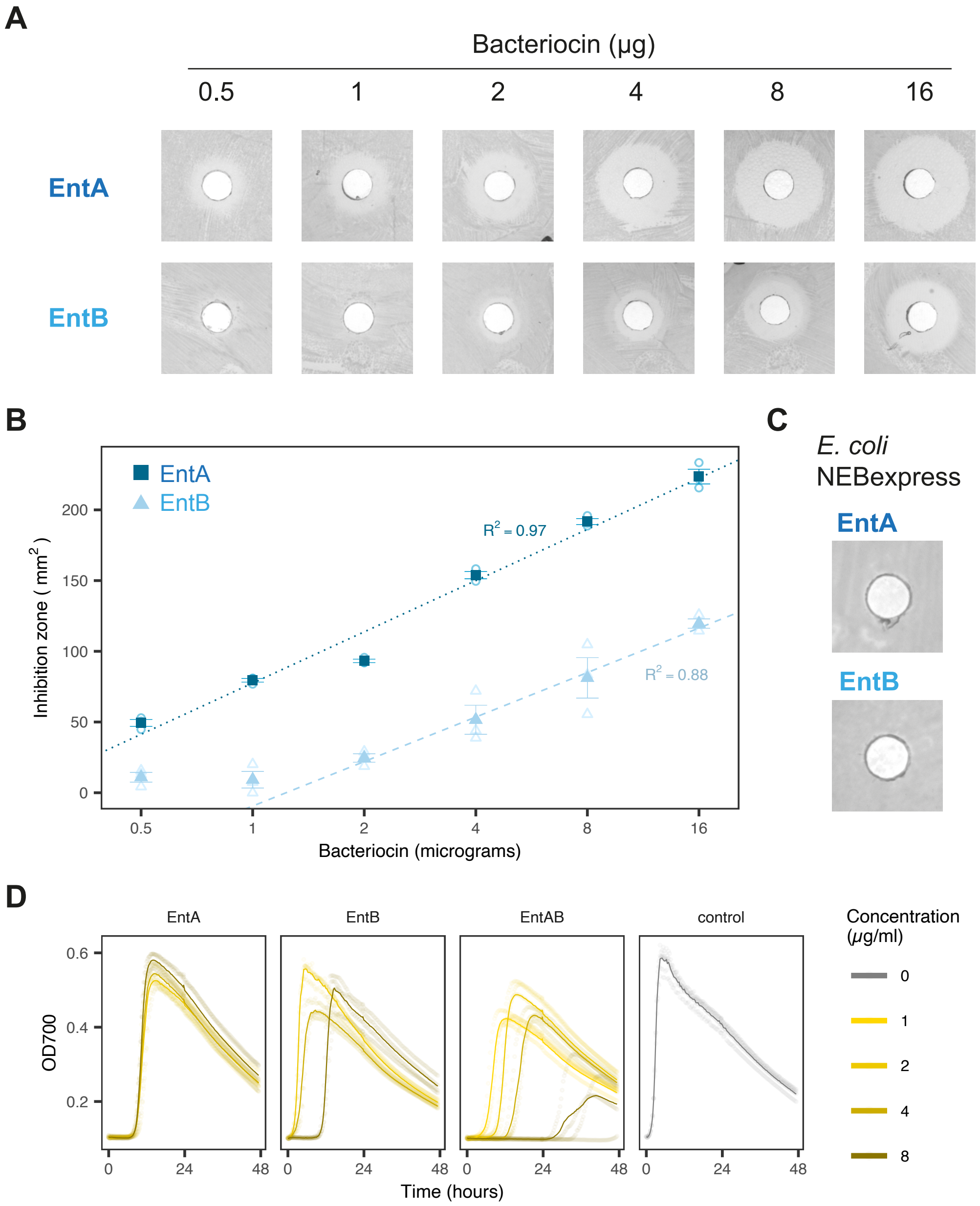

Figure S1: Antimicrobial activity of synthetic EntA and EntB, against *E. faecalis*. (**A**) Representative images of the inhibition zones seen when *E. faecalis* was exposed to varying bacteriocin masses. (**B**) Standard curves of EntA and EntB inhibition zones for different bacteriocin masses. The dashed lines indicate linear regression fits labelled with R^2 values, for EntB the lowest mass was excluded from the linear regression fit as the zone size appeared to plateau ($n = 3$ biological repeats, mean ± SE with individual data points). (**C**) Confirmation that no killing was seen for EntA or EntB against an *E. coli* NEB®express lawn. (**D**) Growth curves of *E. faecalis* grown with the given concentrations of synthetic bacteriocin over 48 hours ($n = 4$ biological repeats, solid lines give mean values).

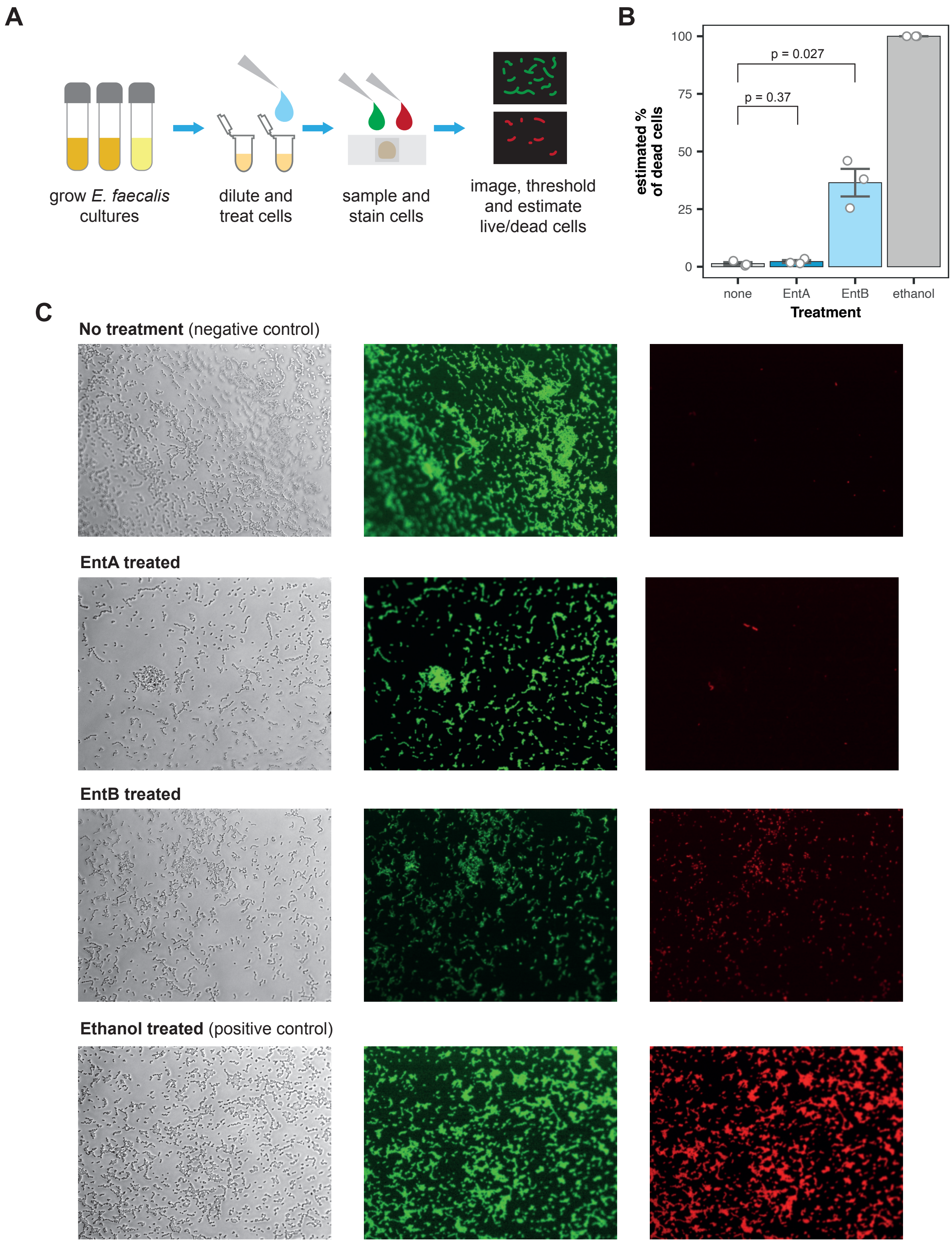

Figure S2: Live/dead assay of synthetic bacteriocin treated *E. faecalis* cells. (**A**) Summary of the live/dead assay protocol. (**B**) Estimates of dead cell percentage for each treatment group. Bars give mean values ± SE, points give individual repeats (n = 3 biological repeats). (**C**) Representative images of each treatment group. Green staining indicates cells, and red staining indicates cell death (left column is brightfield, followed by FITC and TRITC images).

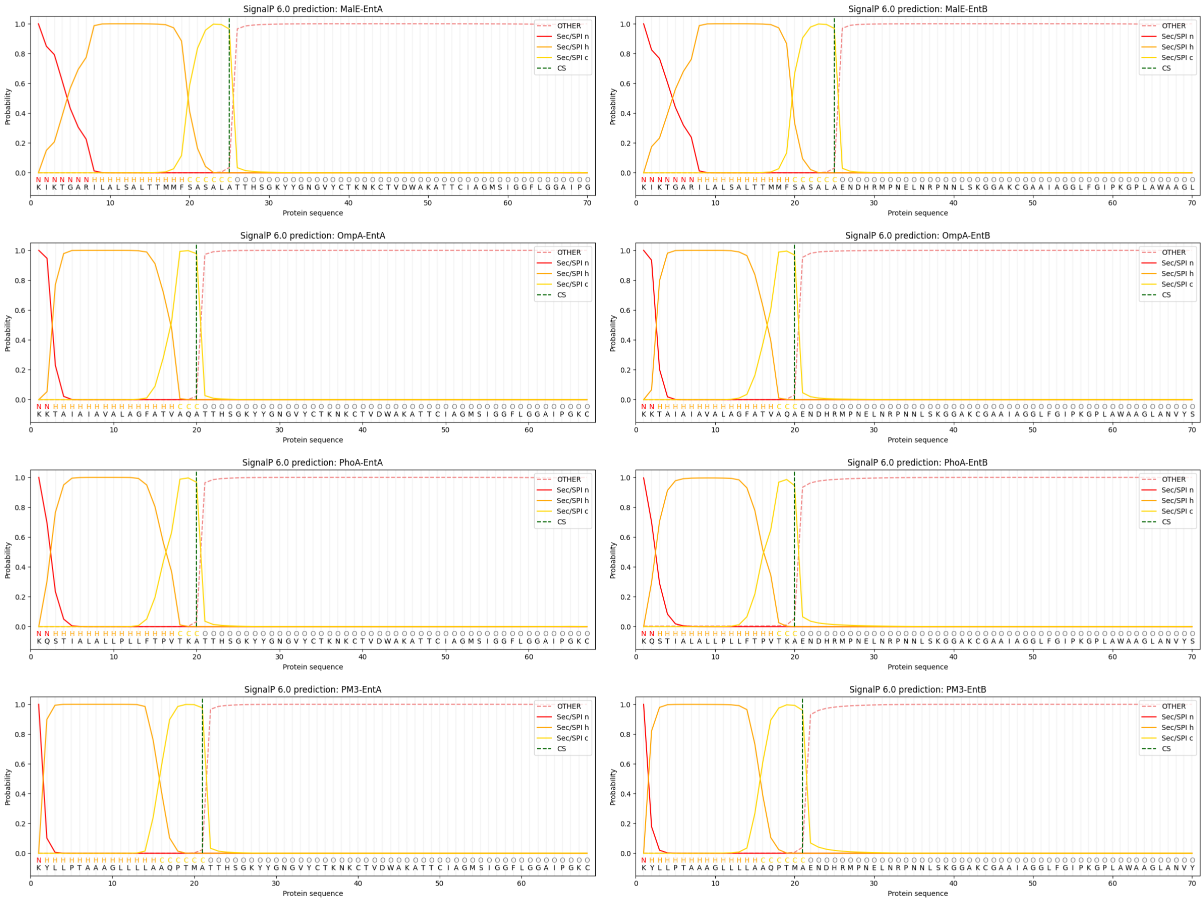

Figure S3: Predictions of the secretion signal locations and cleavage sites, given by the SignalP 6.0 online prediction tool. The predictions are given for all four secretion tags, for EntA (left column) and EntB (right column). SP refers to signal peptide, n denotes the n-region of the peptide, h the h-region and c the c-region. For a full description see Teufel *et al* (2022)[11].

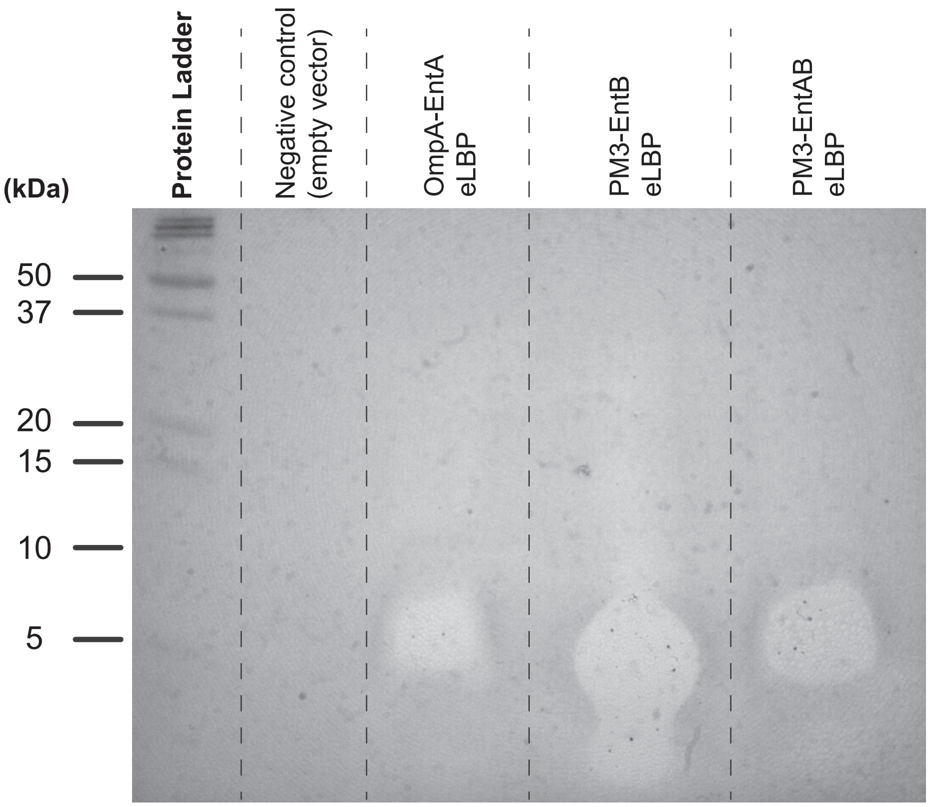

Figure S4: Tricine SDS-PAGE activity gel of concentrated supernatant from *E. coli* NEB®Express with empty vector or eLBP strains expressing OmpA-EntA, PM3-EntB or PM3-EntAB. Ammonium sulphate precipitated supernatant was run on a Tricine SDS-PAGE gel and then transferred to solid growth medium seeded with *E. faecium*. Zones of inhibition corresponded to the expected peptide sizes.

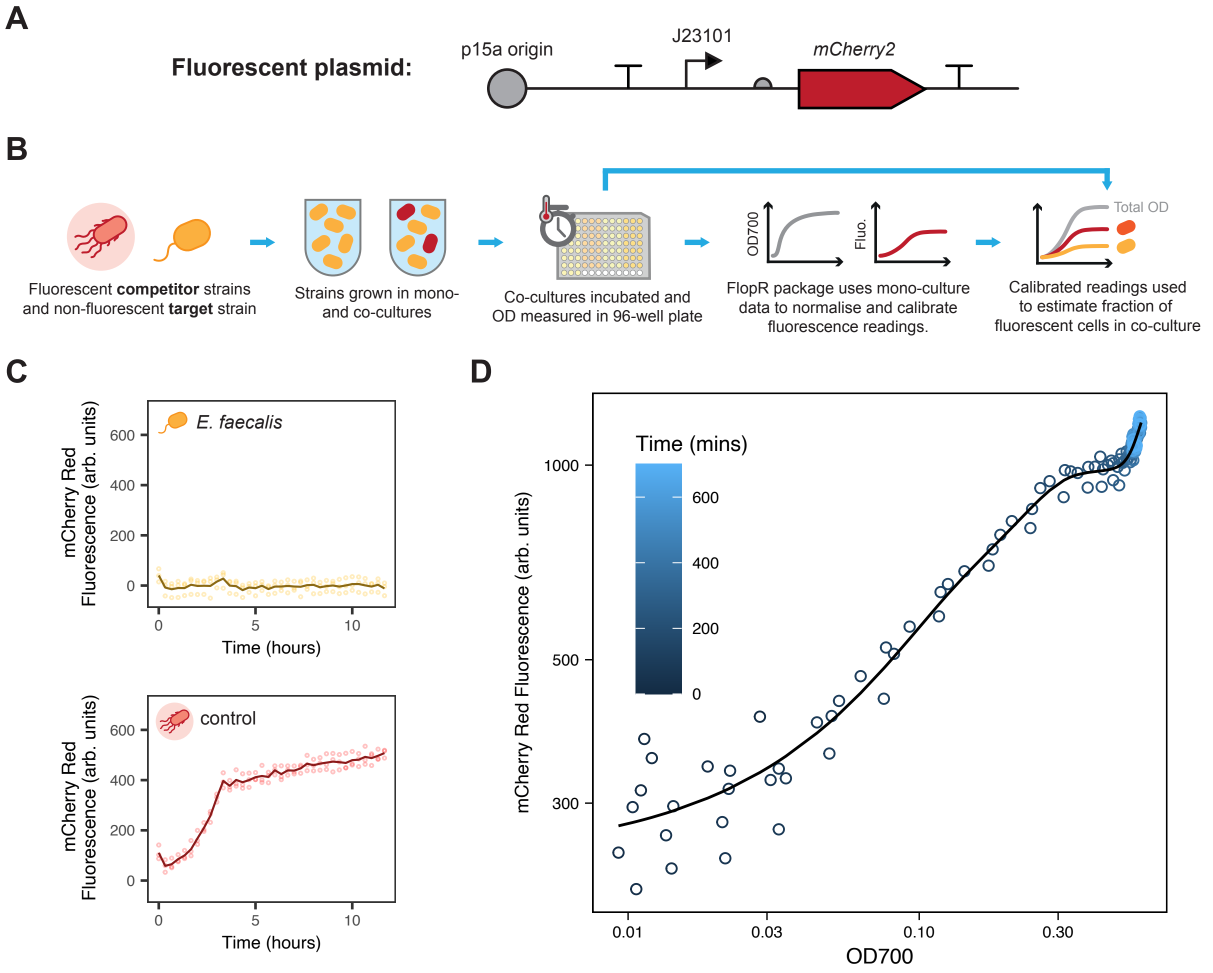

Figure S5: Summary of the FlopR process used to estimate individual species OD in co-cultures. (**A**) Layout of the mCherry2 plasmid used to produce a fluorescent signal in our competitor strains. This is required to perform FlopR analysis on co-culture timecourses. (**B**) Overview of the full FlopR assay protocol. The target and competitor strains are mixed as desired and growth measured in a 96-well plate. The mono-culture of the competitor strain is used to create a calibration curve of expected fluorescent signal for a given OD. From this calibration curve, the fluorescence measured in co-culture can be used to estimate the ratio of each species present. This ratio is used to estimate a value for the OD of each species in the co-culture. (**C**) Fluorescent mCherry signal from the target *E. faecalis* and control strain over time. It can be seen that the *E. faecalis* produces minimal fluorescence and therefore, does not interfere with the FlopR calibration process (n = 3 biological repeats). (**D**) A representative fluorescence vs OD_{700} calibration curve. This curve can be used to estimate the species OD for a competitor strain from a given fluorescent signal. For a more detailed description see Fedorec *et al* (2020)[12].

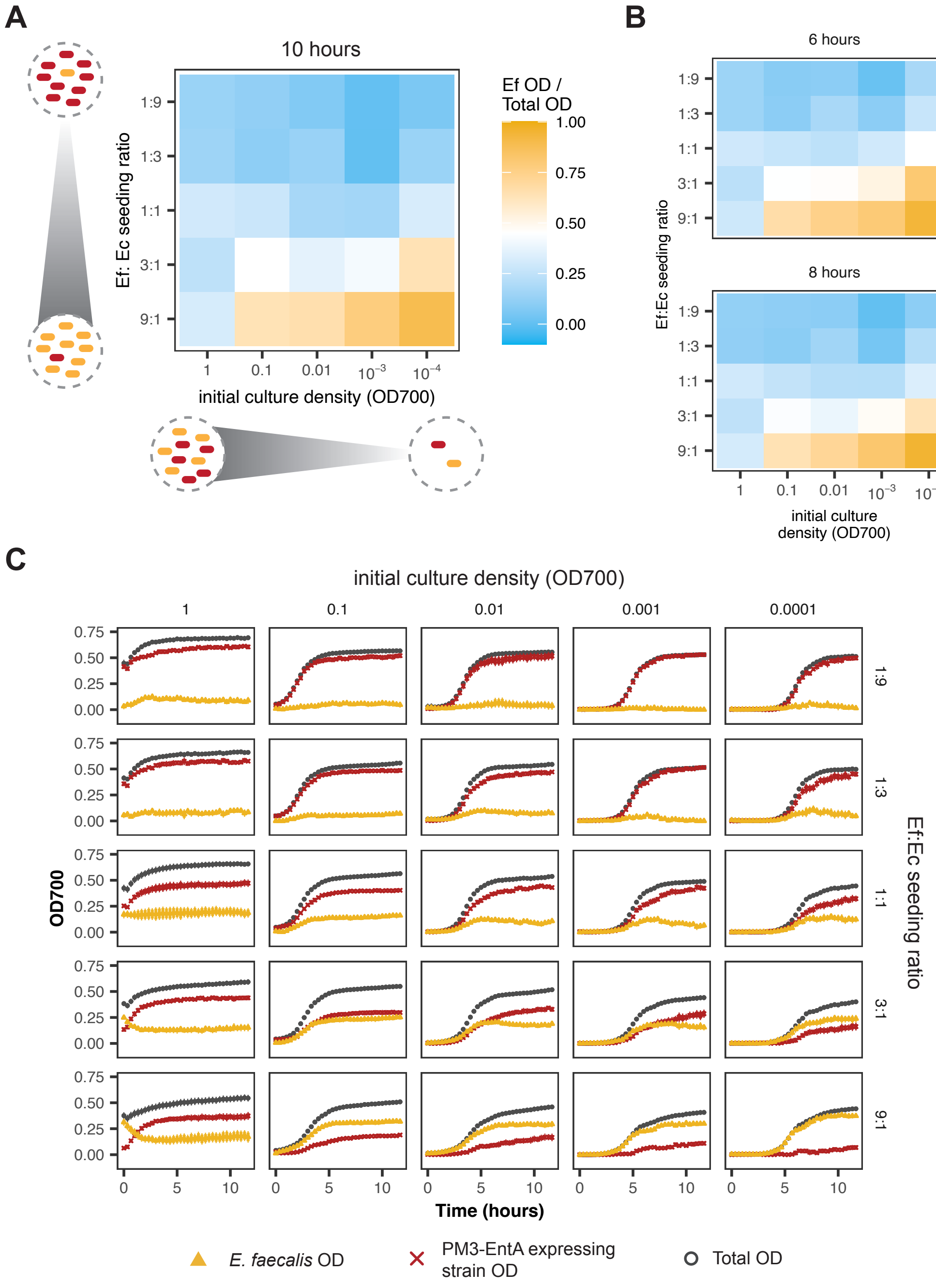

Figure S6: The effect of seeding ratio and starting density on co-culture growth. (**A**) The ten hour ratio of estimated *E. faecalis* OD over total OD for the given starting culture densities and seeding ratios. (**B**) The same estimated ratios for the 6 hour and 8 hour timepoints. (**C**) The growth curves of co-cultures from each starting condition, orange and red lines show the estimated *E. faecalis* and competitor OD, respectively (n = 3 biological repeats, lines give mean ± SE).

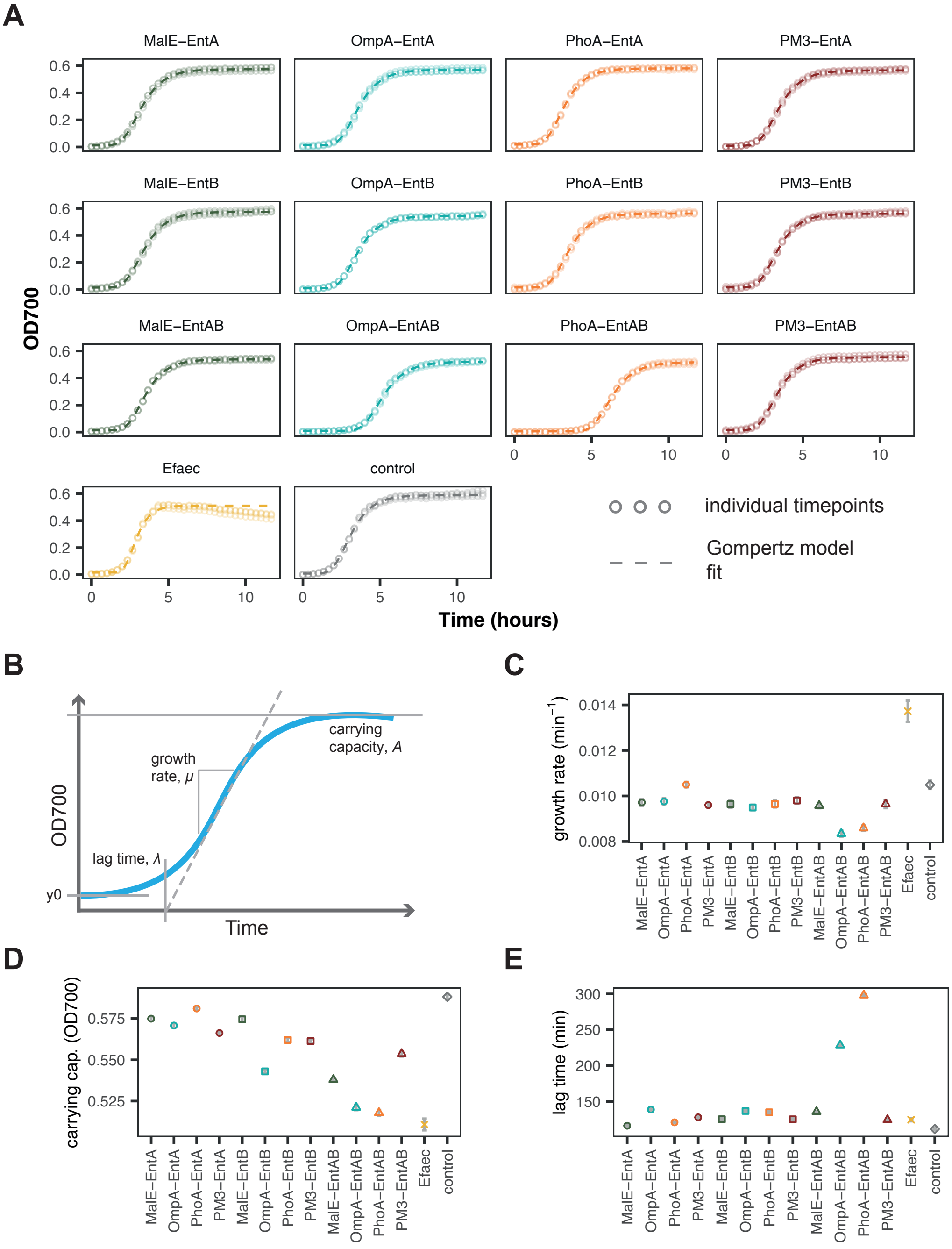

Figure S7: (**A**) Growth curves of strains grown in mono-culture, fitted with the Gompertz model. Panel labels give the respective strains, 'Efaec' refers to *E. faecalis*. Fitting of the *E. faecalis* growth curve was trimmed to 400 minutes, to exclude the effect of dropping OD700 at later timepoints (n = 3 biological repeats, dashed lines give model fit and points individual repeats). Parameters from Gompertz model fits of the mono-culture growth curves, strains are labelled based on the bacteriocin construct they expressed: (**B**) Diagram of a typical growth curve illustrating the fitted Gompertz parameters. The fitted Gompertz parameter values for (**C**) growth rate, (**D**) carrying capacity and (**E**) lag time (fitted value ± fitted error).

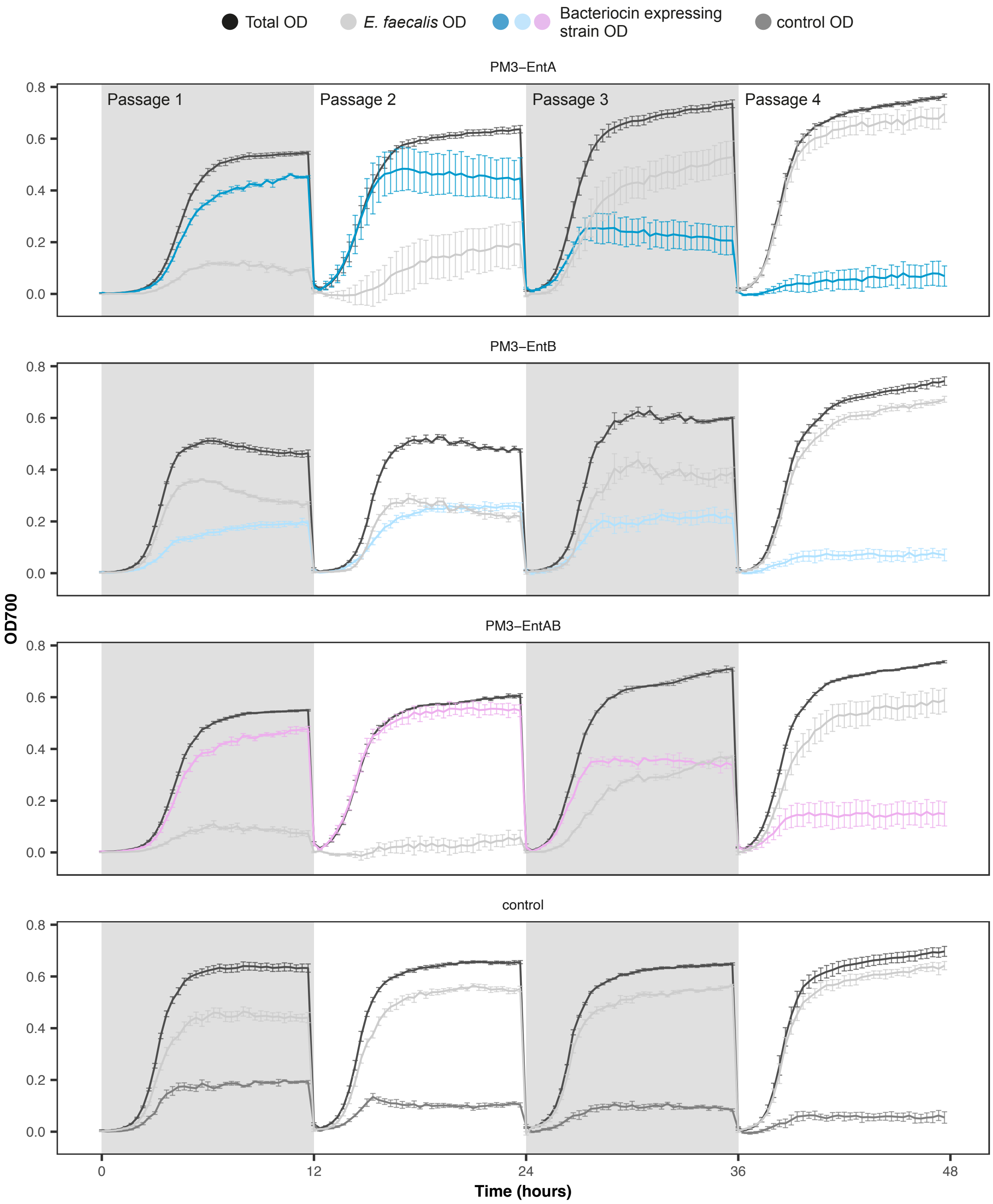

Figure S8: Co-culture time series of *E. faecalis* grown in co-culture with each of the labelled strains. Co-cultures were passaged every 12 hours, for a total of 48 hours. For all co-cultures *E. faecalis* growth increases over the 48 hour period, indicating a reduction in antimicrobial activity. Anomalous datapoints were excluded from the averages, shaded areas indicate each passage (n = 4 biological repeats, mean values ± SE).

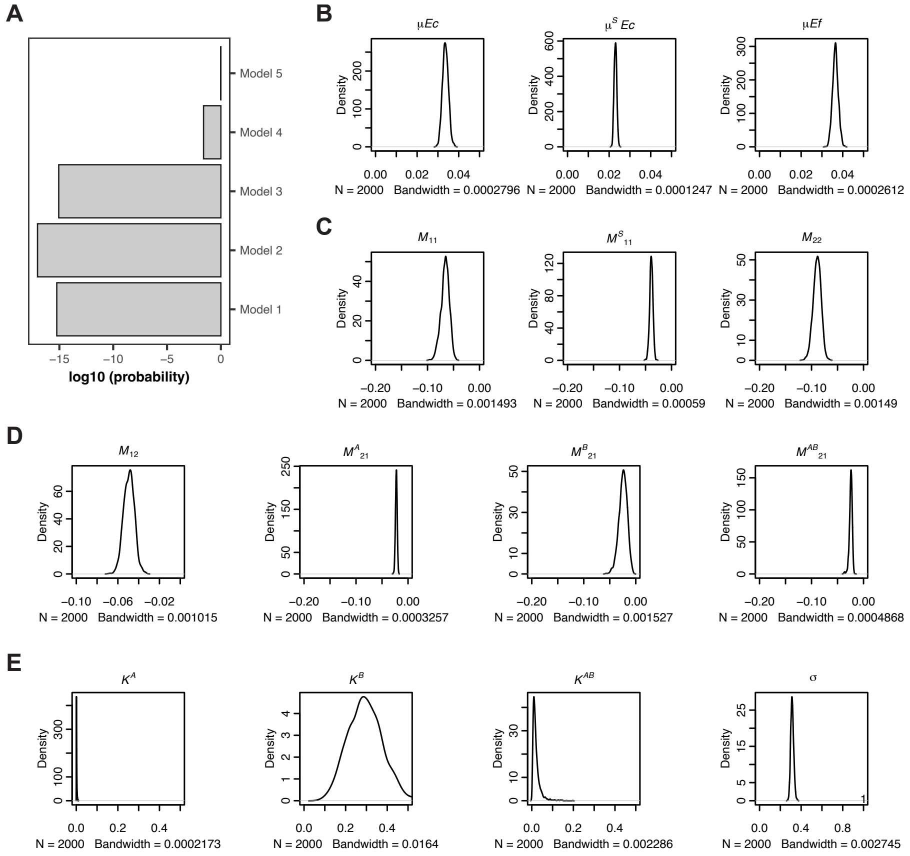

Figure S9: (**A**) The probability of each of the five models explored here. Posterior distributions for each of the parameters, calculated during Bayesian fitting of model 5: (**B**) species growth rates, (**C**) self-interaction terms, (**D**) interspecies interaction terms and (**E**) the K^s and σ parameters.

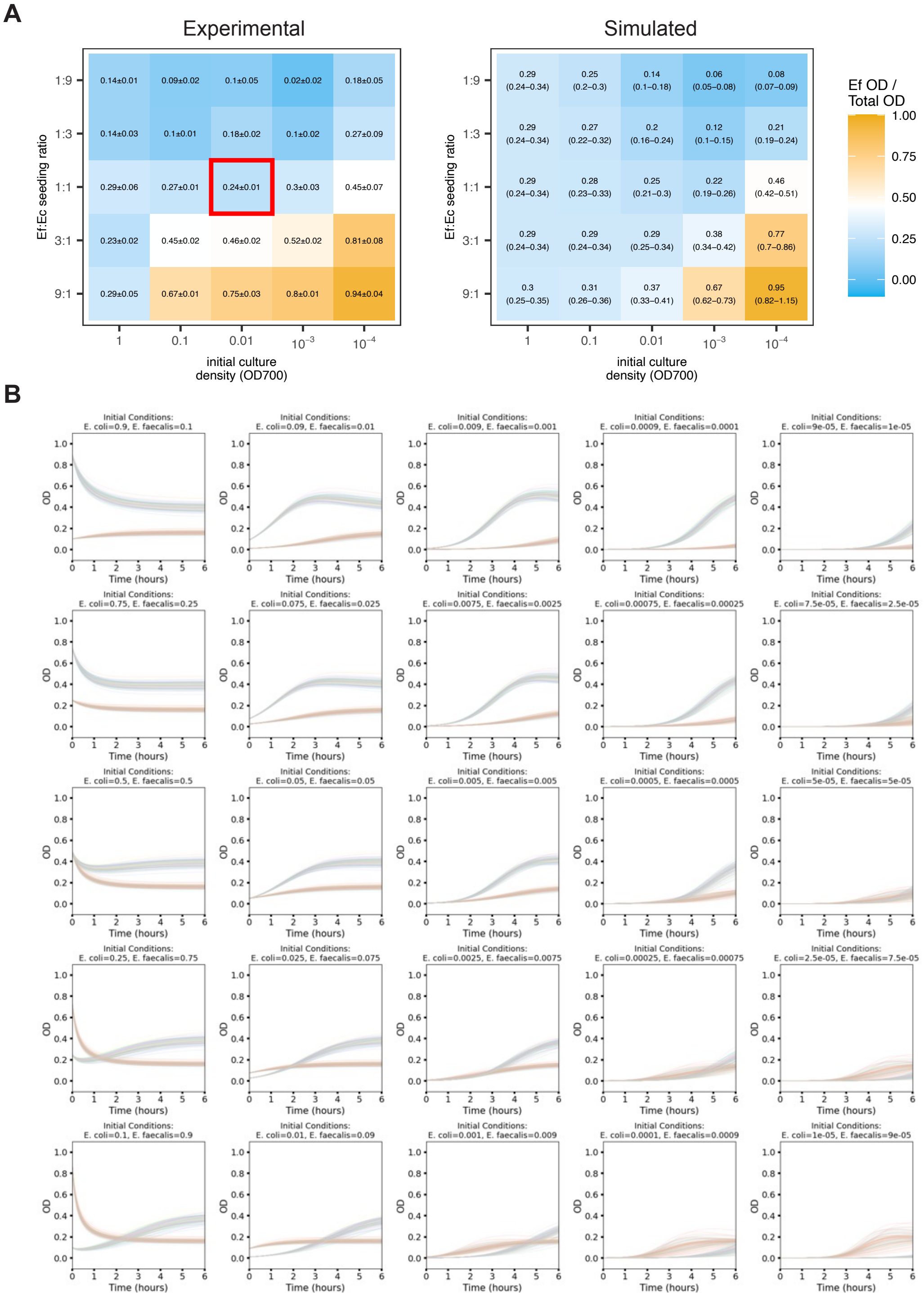

Figure S10: (**A**) Comparison of the ratios of *E. faecalis* over total OD at 6 hours for the experimental system (left panel, mean ± SE, taken from Figure S6) against the simulated model (right panel, median and 0.95 credible regions). The model was fit using only data from the experimental condition highlighted in red. (**B**) Simulated timecourses of *E. coli* PM3-EntA (blue line) and *E. faecalis* (orange line) co-cultures over 6 hours, using the developed model. The starting conditions for each simulation are given in the panel labels.

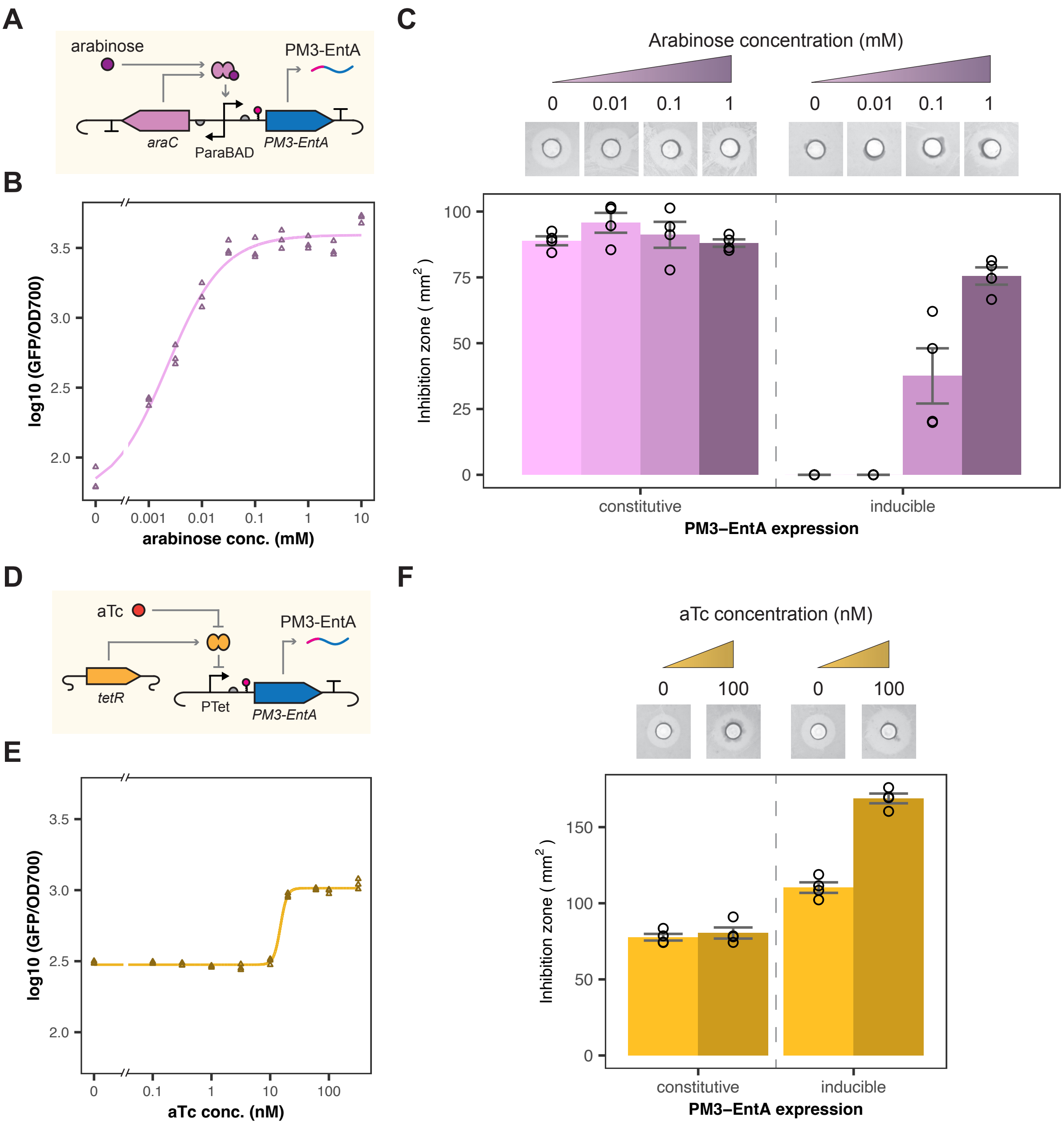

Figure S11: Design and characterisation of an arabinose (**A** - **C**) and aTc (**D** - **F**) inducible bacteriocin producing strains. Plasmid designs of the arabinose-inducible (**A**) and aTc-inducible (**D**) circuits. GFP response of the arabinose (**B**) and aTc (**E**) inducible circuits in liquid culture (n = 3 biological repeats, median values fitted with a Hill Function). Inhibition zone assays of the PM3-EntA constitutive and arabinose-inducible strains (**C**) or the PM3-EntA constitutive and aTc-inducible strains (**F**), for the given inducer concentrations. Insets show representative images of the inhibition zones (n = 4 biological repeats, mean values ± SE).

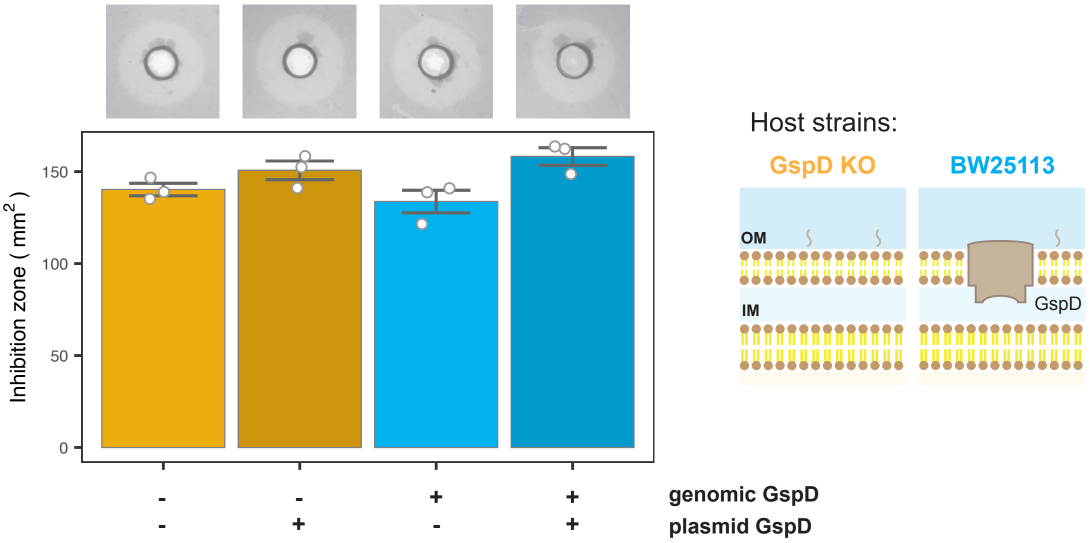

Figure S12: Inhibition assays of the PM3-EntA and PM3-EntA-GspD constructs, expressed from two host strains: the $\Delta gspD$ knockout (yellow bars), and $gspD^{+}$ BW25113 (blue bars) strains. The x-axis labels indicate plasmid and genomic expression of GspD. GspD pore deletion did not prevent bacteriocin killing under the conditions tested. Insets show representative images of the inhibition zones (n = 3 biological repeats, mean values ± SE).

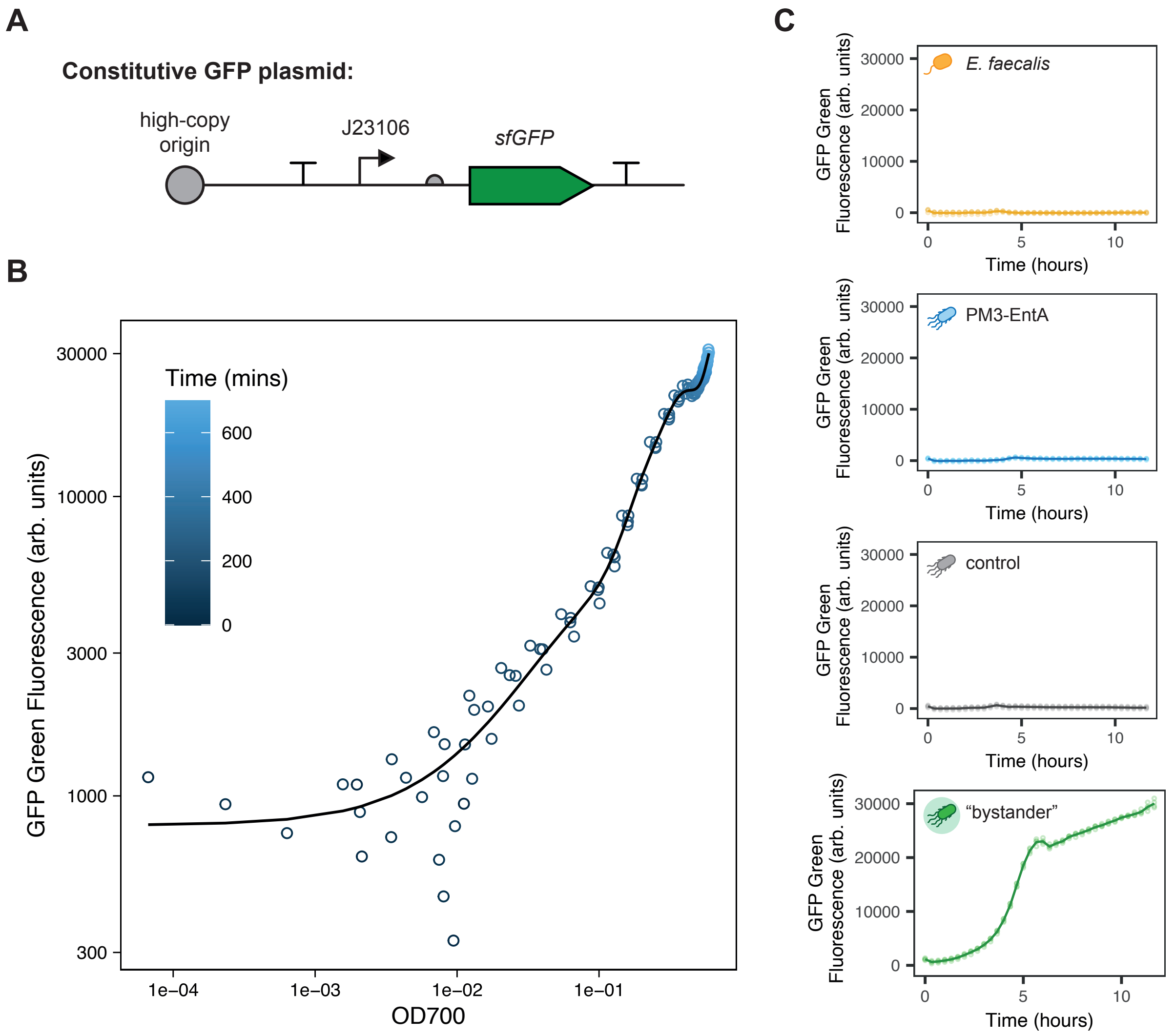

Figure S13: Summary of the incorporation of a (GFP-fluorescent) bystander strain into the liquid-culture assays. (**A**) Layout of the GFP plasmid used to produce a fluorescent signal in our bystander strain. (**B**) A representative fluorescence vs OD_{700} calibration curve, used to estimate the species OD of the bystander strain. (**C**) Fluorescent GFP signal from the *E. faecalis*, PM3-EntA, control and bystander strains over time. Only the bystander strain was found to produce a strong GFP signal (n = 3 biological repeats.

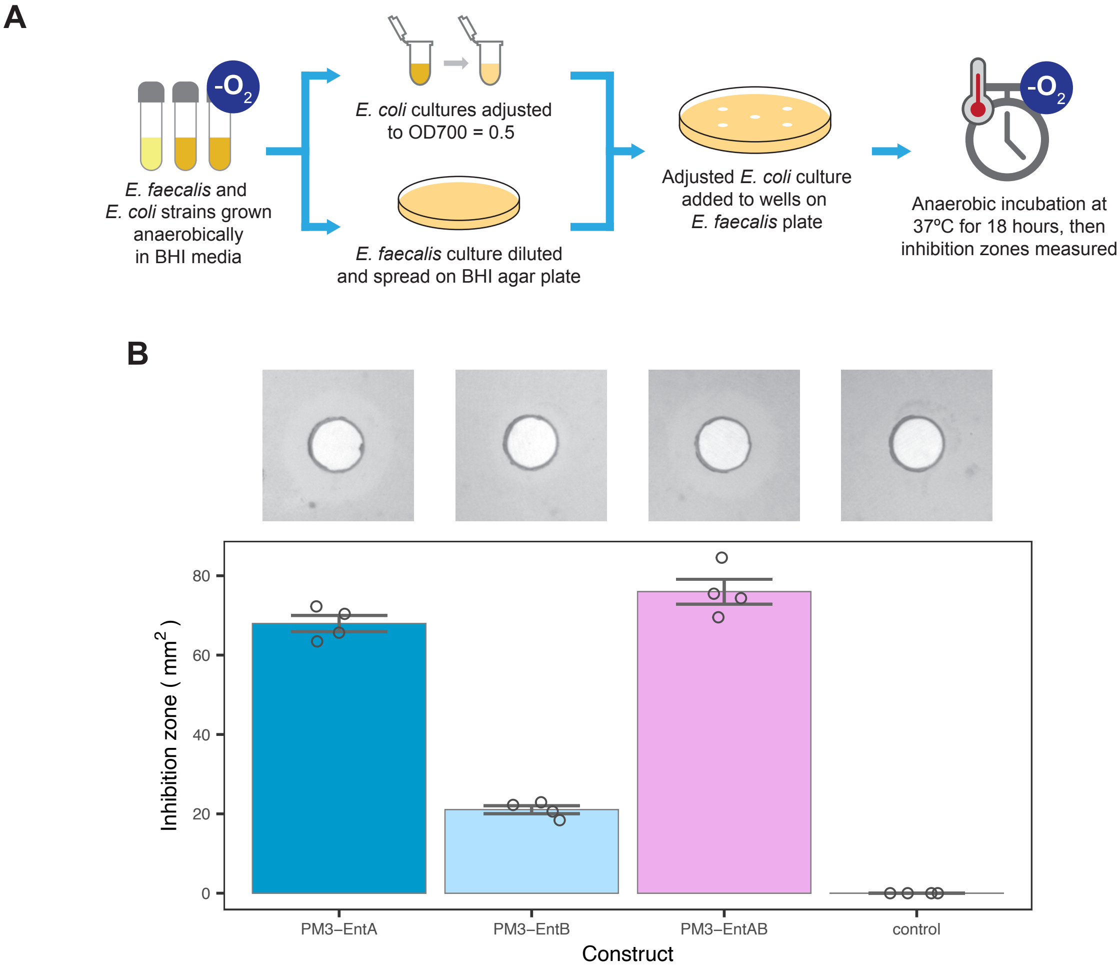

Figure S14: Inhibition zones of the PM3-bacteriocin expressing strains, under anaerobic conditions. (**A**) Overview of the anaerobic assay protocol. All cultures were grown in the absence of oxygen. (**B**) Inhibition zone assay of the PM3-bacteriocin constructs under anaerobic conditions. Inhibition zones were seen for all bacteriocin producing strains tested. Insets show representative images of the inhibition zones observed (n = 4 biological repeats, bars indicate mean ± SE).

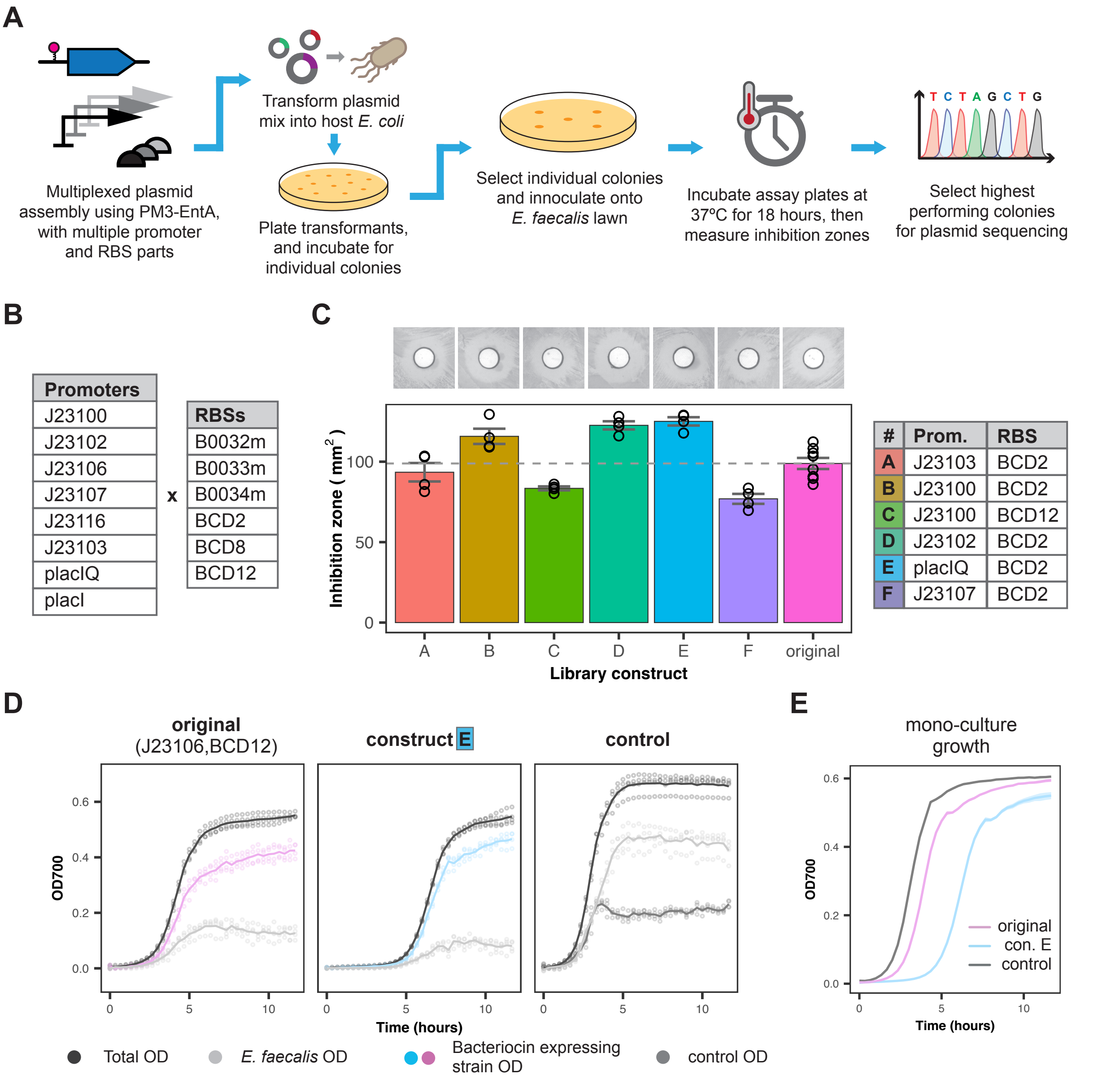

Figure S15: Randomised library of PM3-EntA expressing constructs. (**A**) The cloning workflow used to generate the construct library. (**B**) The promoter and RBS parts included in the MoClo reaction. (**C**) Solid-culture characterisation of six strains compared against the original PM3-EntA strain, reported in the main manuscript, as a positive control. The table gives the respective promoter and RBS parts in each construct (n = 4 biological repeats for new constructs, n = 8 biological repeats for original construct, bars indicate mean ± SE). (**D**) Liquid co-culture characterisation of construct E versus the original PM3-EntA and control strains (n = 4 biological repeats, lines indicate means and points show individual repeats). (**E**) Growth curves of construct E, the original PM3-EntA and control strains (n = 4 biological repeats, lines indicate mean ± SE). Construct E was found to have impaired growth compared to the original PM3-EntA and control strains.

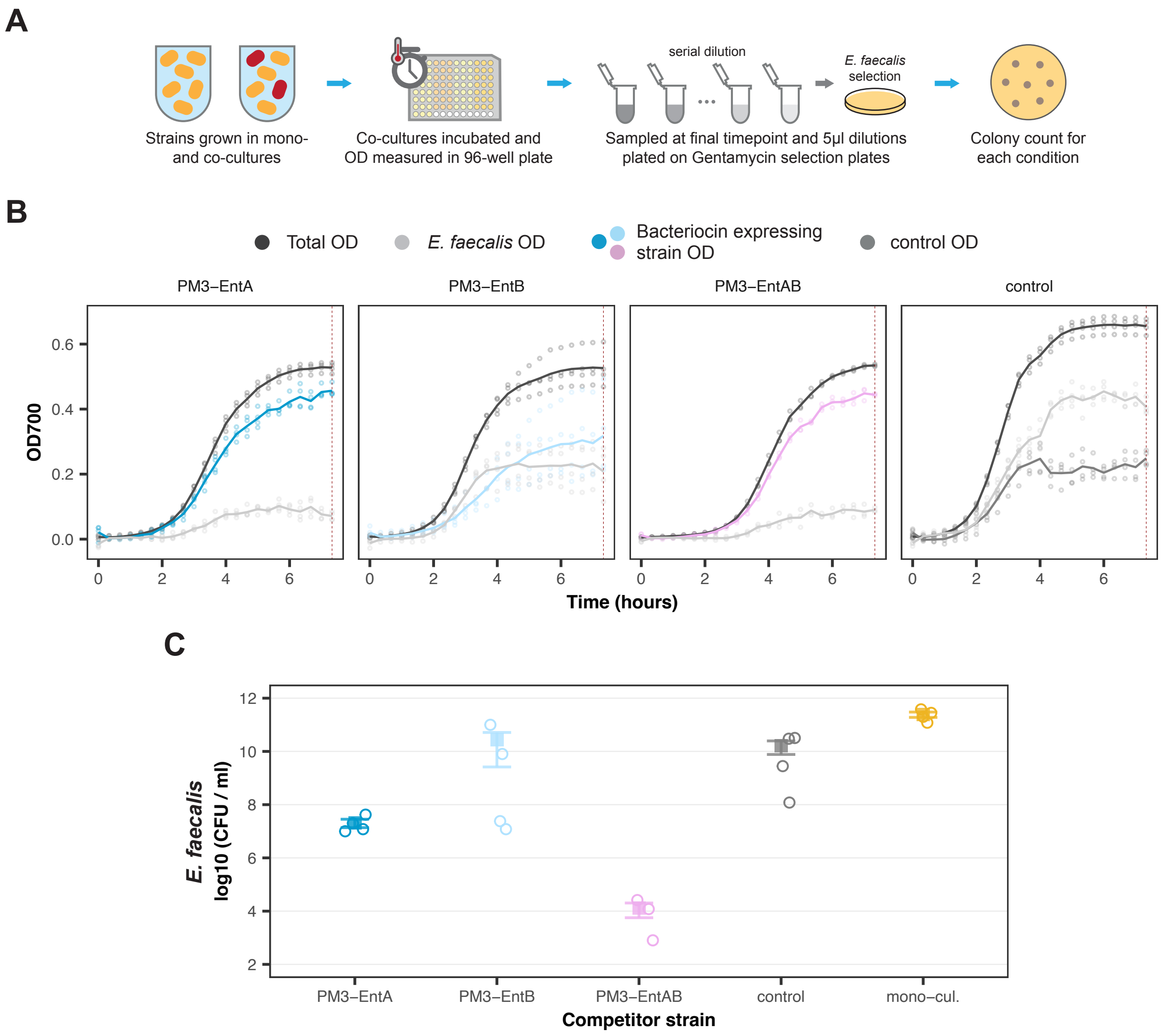

Figure S16: Colony counts of *E. faecalis* growth from FlopR co-culture assays. (**A**) The experimental protocol used for collecting estimates of colony counts from the FlopR co-culture assays. In brief, strains were prepared and grown in co-culture as described in Figure 3A. After 8 hours of growth, all co-cultures were sampled and serial dilutions plated on gentamycin containing media to select for *E. faecalis* growth. (**B**) Timecourses of the FlopR co-cultures sampled for colony counting, the red dashed line indicates the time at which samples were taken for plating (n = 4 biological repeats, n = 3 biological repeats for PM3-EntAB co-cultures, solid lines give mean values). (**C**) The estimated colony counts of *E. faecalis* for each of the given culture conditions (n= 4 biological repeats, n = 3 biological repeats for PM3-EntAB co-cultures, mean values ± SE).

References

[1] Tomoya Baba, Takeshi Ara, Miki Hasegawa, Yuki Takai, Yoshiko Okumura, Miki Baba, Kirill A Datsenko, Masaru Tomita, Barry L Wanner, and Hirotada Mori. Construction of *Escherichia coli* K-12 in-frame, single-gene knockout mutants: the Keio collection. *Molecular Systems Biology*, 2, 1 2006.

[2] Adam J. Meyer, Thomas H. Segall-Shapiro, Emerson Glassey, Jing Zhang, and Christopher A. Voigt. Escherichia coli Marionette strains with 12 highly optimized small-molecule sensors. *Nature Chemical Biology*, 15(2):196–204, February 2019.

[3] Samuel O Skinner, Leonardo A Sepúlveda, Heng Xu, and Ido Golding. Measuring mRNA copy number in individual *Escherichia coli* cells using single-molecule fluorescent in situ hybridization. *Nature Protocols*, 8(6):1100–1113, June 2013.

[4] Johannes Schindelin, Ignacio Arganda-Carreras, Erwin Frise, Verena Kaynig, Mark Longair, Tobias Pietzsch, Stephan Preibisch, Curtis Rueden, Stephan Saalfeld, Benjamin Schmid, Jean-Yves Tinevez, Daniel James White, Volker Hartenstein, Kevin Eliceiri, Pavel Tomancak, and Albert Cardona. Fiji: an open-source platform for biological-image analysis. *Nature Methods*, 9(7):676–682, July 2012.

[5] Sonya V. Iverson, Traci L. Haddock, Jacob Beal, and Douglas M. Densmore. Cidar moclo: Improved moclo assembly standard and new *E. coli* part library enable rapid combinatorial design for synthetic and traditional biology. *ACS Synthetic Biology*, 5:99–103, 1 2016.

[6] Babak Momeni, Li Xie, and Wenying Shou. Lotka-Volterra pairwise modeling fails to capture diverse pairwise microbial interactions. *Elife*, 6:e25051, 2017.

[7] Vanni Bucci, Belinda Tzen, Ning Li, Matt Simmons, Takeshi Tanoue, Elijah Bogart, Luxue Deng, Vladimir Yeliseyev, Mary L Delaney, Qing Liu, et al. Mdsine: Microbial dynamical systems inference engine for microbiome time-series analyses. *Genome biology*, 17:1–17, 2016.

[8] Chitong Rao, Katharine Z Coyte, Wayne Bainter, Raif S Geha, Camilia R Martin, and Seth Rakoff-Nahoum. Multi-kingdom ecological drivers of microbiota assembly in preterm infants. *Nature*, 591(7851):633–638, 2021.

[9] Stan Development Team. RStan: the R interface to Stan, 2023. R package version 2.21.8.

[10] Bob Carpenter, Andrew Gelman, Matthew D Hoffman, Daniel Lee, Ben Goodrich, Michael Betancourt, Marcus Brubaker, Jiqiang Guo, Peter Li, and Allen Riddell. Stan: A probabilistic programming language. *Journal of statistical software*, 76(1), 2017.

[11] Felix Teufel, Jos Juan Almagro Armenteros, Alexander Rosenberg Johansen, Magns Halldr Gslason, Silas Irby Pihl, Konstantinos D. Tsirigos, Ole Winther, Sren Brunak, Gunnar von Heijne, and Henrik Nielsen. SignalP 6.0 predicts all five types of signal peptides using protein language models. *Nature Biotechnology*, 40:1023–1025, 7 2022.

[12] Alex J. H. Fedorec, Clare M. Robinson, Ke Yan Wen, and Chris P. Barnes. FlopR: An open source software package for calibration and normalization of plate reader and flow cytometry data. *ACS Synthetic Biology*, 9:2258–2266, 9 2020.
